# Two-Step Chemo-Microbial Degradation of Post-Consumer Polyethylene Terephthalate (PET) Plastic Enabled by a Biomass-Waste Catalyst

**DOI:** 10.3390/bioengineering10111253

**Published:** 2023-10-26

**Authors:** Deepika Shingwekar, Helen Laster, Hannah Kemp, Jay L. Mellies

**Affiliations:** Department of Biology, Reed College, Portland, OR 97202, USA; dshingwekar@reed.edu (D.S.); lasterh@gmail.com (H.L.); hannah.mk425@gmail.com (H.K.)

**Keywords:** polyethylene terephthalate, PET plastic, depolymerization, biocatalyst, biodegradation, glycolysis, recycling

## Abstract

Polyethylene terephthalate (PET) pollution has significant environmental consequences; thus, new degradation methods must be explored to mitigate this problem. We previously demonstrated that a consortium of three *Pseudomonas* and two *Bacillus* species can synergistically degrade PET in culture. The consortium more readily consumes bis(2-hydroxyethyl) terephthalate (BHET), a byproduct created in PET depolymerization, compared to PET, and can fully convert BHET into metabolically usable monomers, namely terephthalic acid (TPA) and ethylene glycol (EG). Because of its crystalline structure, the main limitation of the biodegradation of post-consumer PET is the initial transesterification from PET to BHET, depicting the need for a transesterification step in the degradation process. Additionally, there have been numerous studies done on the depolymerization reaction of PET to BHET, yet few have tested the biocompatibility of this product with a bacterial consortium. In this work, a two-step process is implemented for sustainable PET biodegradation, where PET is first depolymerized to form BHET using an orange peel ash (OPA)-catalyzed glycolysis reaction, followed by the complete degradation of the BHET glycolysis product by the bacterial consortium. Results show that OPA-catalyzed glycolysis reactions can fully depolymerize PET, with an average BHET yield of 92% (*w*/*w*), and that the reaction product is biocompatible with the bacterial consortium. After inoculation with the consortium, 19% degradation of the glycolysis product was observed in 2 weeks, for a total degradation percentage of 17% when taking both steps into account. Furthermore, the 10-week total BHET degradation rate was 35%, demonstrating that the glycolysis products are biocompatible with the consortium for longer periods of time, for a total two-step degradation rate of 33% over 10 weeks. While we predict that complete degradation is achievable using this method, further experimentation with the consortium can allow for a circular recycling process, where TPA can be recovered from culture media and reused to create new materials.

## 1. Introduction

It is currently estimated that 80% of plastic ever made ends up in landfills or as pollution within the environment [1]. If the present trends continue, approximately 12,000 million metric tons of plastic will be discarded in this way by 2050, and roughly half of these plastics are single-use materials. Due to the increased demand for sterile, single-use plastic during the COVID-19 pandemic, the plastic pollution crisis shows no sign of slowing down [2,3]. The diverse applications and durability of plastics allow these materials to be produced at a rapid rate, and current efforts to mitigate the environmental effects of pollution fall short. Polyethylene terephthalate (PET) is one of the most extensively produced polymers, with applications in bottles, packaging, textiles, and more [4].

The most common PET recycling methods can be categorized into energy recovery, mechanical recycling, and chemical recycling. Energy recovery and mechanical recycling both produce low-quality materials lacking in durability [5]. The chemical recycling of PET involves the depolymerization of PET into monomers, namely terephthalic acid (TPA) and ethylene glycol (EG), or the hydrolysis products bis(2-hydroxyethyl) terephthalate (BHET) and 2-hydroxyethyl terephthalic acid (MHET). Novel sustainable catalytic methods behind the solvolysis, or glycolysis, of PET use biomass-waste-derived catalysts, like orange peel ash (OPA). These procedures produce BHET in similar yields to protocols using traditional catalysts like heavy metal acetate salts [6,7,8,9,10,11,12]. Chemical recycling is primarily focused on reproducing materials using monomers released through depolymerization but involves additional separation and purification steps to yield high-quality monomers. These complex and resource-intensive procedures lead to higher production costs and reduced overall profitability compared to traditional mechanical recycling methods.

To address the environmental issues of energy recovery and the mechanical and chemical recycling of PET, the use of microbial and enzymatic degradation has been explored in recent years to provide a renewable and more natural solution. Enzymes found in microorganisms that are able to degrade naturally occurring biopolymers, such as lignin, that are chemically similar to synthetic polymers have been isolated and applied to plastic recycling with varying success [13,14]. Out of all the polymers, there has been a concerted effort to identify and improve PET-degrading enzymes and microbes [14,15,16]. Specifically, we previously isolated a microbial consortium consisting of five *Pseudomonas* and *Bacillus* spp. that showed the synergistic degradation of PET [17]. The bacteria, isolated from petroleum-polluted soils, encode 250 enzymes known to degrade biopolymers, synthetic polymers, and plasticizers [18]. Cross-feeding, in part, explained the ability of the bacteria to synergistically degrade PET plastic [19,20,21].

As a means to increase the applicability of chemical and biological recycling, chemo-enzymatic PET degradation processes have been developed to produce high-quality monomers without the time- and resource-intensive steps [22,23,24,25]. These processes follow the pattern of chemical depolymerization, enzymatic hydrolysis, and monomer extraction, with high efficiency, and they show promise as an environmentally friendly alternative to conventional plastic recycling. Despite the improvement in terms of chemical recycling product purification, purified enzymes have limited stability and are costly and time-consuming to maintain. 

**Scheme 1:**
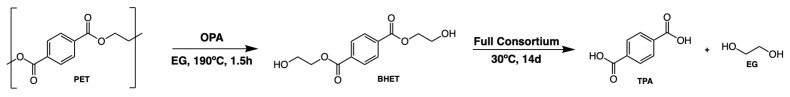
Chemo-microbial degradation pathway.

Another important consideration is that, to date, there is no known enzyme that degrades high-crystallinity PET plastic, which is approximately 20% crystalline. Rather, most studies have used low-crystallinity PET film, ~2% to 5%, to study this process [21,26]. Therefore, semi-microbial degradation pathways are a better choice for the biodegradation of highly crystalline PET water bottles. Thus, this work proposes a chemo-microbial approach for the complete degradation of post-consumer PET, using OPA-catalyzed glycolysis for chemical depolymerization and a microbial consortium containing intact bacteria for degradation, as seen in Scheme 1. Bacterial degradation, rather than the more standard enzymatic hydrolysis, bypasses some of the limitations associated with traditional enzymatic methods and offers a more reproducible and less expensive alternative. Coupled with chemical depolymerization, the two-step degradation pathway allows for a more applicable approach to post-consumer PET recycling using biological methods. 

## 2. Materials and Methods

### 2.1. Materials

All reagents, except for the oranges and PET flakes, were purchased from Sigma-Aldrich (Sigma-Aldrich, St. Louis, MO, USA) and used without further purification. Oranges were obtained from local vendors in Portland, OR, USA. Empty 2 L PepsiCo PET bottles were collected, and caps and labels were removed. The PET bottles were then washed thoroughly with deionized (DI) H_2_O, dried in the open air, and shredded in a commercial blender to produce the PET flakes used in the glycolysis reaction. 

### 2.2. Preparation of OPA Catalyst

The preparation of the OPA catalyst was adapted from Laldinpuii et al., 2021 [7]. After the fruit was peeled, the orange peels were washed thoroughly and dried in an oven at 50 °C for 7 days. The dried peels were burned in the open air using a blowtorch and then sieved through a 100 BS sieve to remove any larger particulates. OPA was stored in a tightly sealed container at room temperature and was characterized through FTIR-ATR, PXRD, and ICP-MS. 

### 2.3. PET Glycolysis

PET was depolymerized using an OPA-catalyzed glycolysis reaction adapted from Lalhmangaihzuala et al., 2020 [12]. PET flakes (10 g, 52.03 mmol) were reacted with EG (50 g, 805.50 mmol) and OPA (1 g) at 190 °C for 1.5 h. The reaction mixture was vacuum-filtered and then washed with 450 mL DI H_2_O heated to 60 °C to separate the remaining catalyst. The filtrate was stirred vigorously for 45 min at 60 °C to avoid BHET crystallization as it is soluble in water at this temperature. The solution was filtered using Whatman Grade 1 Qualitative Filter Paper (11 µm pore size) to remove any water-insoluble solid, predicted to be oligomers or other byproducts (11.81% yield), and characterized through NMR, FTIR-ATR, HPLC, and XRD. The filtrate was then refrigerated at 4 °C for 12 h for crystallization. The crystallized BHET product was obtained (78.40% yield) through filtration, air-dried, and characterized through NMR, FTIR-ATR, HPLC, and XRD. The BHET monomer yield was determined through Equation (1):

(1) $$Yield\;of\;BHET\;monomer\;\left(\%\right)=\frac{W_{BHET}}{W_{PET}}\times \frac{{MW}_{PET}}{{MW}_{BHET}}\times 100$$

where *W_BHET_*, *W_PET_*, *MW_BHET_*, and *MW_PET_* refer to the weight of the *BHET* produced, the weight of the initial *PET*, the molecular weight of *BHET* (254.24 g mol^−1^), and the molecular weight of *PET* (192.2 g mol^−1^), respectively. Under-polymerized *PET* flakes were washed and weighed to determine *PET* conversion, through Equation (2):

(2) $$Conversion\;of\;PET\;\left(\%\right)=\frac{W_{PET,I}-W_{PET,U}}{W_{PET,I}}$$

where *W_PET_*_,*I*_ and *W_PET_*_,*U*_ refer to the weight of the initial *PET* and the under-polymerized *PET*, respectively. 

### 2.4. Characterization of Glycolysis Products and OPA Catalyst

#### 2.4.1. Nuclear Magnetic Resonance (NMR)

The 1-D ^1^H-NMR and ^13^C-NMR were recorded using a 400 MHz Bruker Avance I spectrometer equipped with an auto-tune and match (ATM) broadband probe and an autosampler. Approximately 25 mg of product was dissolved in 600 μL of DMSO-d6 and filtered through a 0.22 μm PES syringe filter to create each sample. The samples were transferred to Wilmad 5-mm-diameter precision-grade NMR tubes and analyzed. Spectra were visualized and analyzed using the MestReNova (Mnova) software version 3.15 Build 267. The chemical shifts were compared to the BHET reference spectra found in Ghaemy et al., 2005 [27]. 

#### 2.4.2. Fourier-Transform Infrared Spectroscopy–Attenuated Total Reflectance (FTIR-ATR)

FT-IR spectra were obtained using a Thermo iS5 FT-IR Spectrometer with an iD7 ATR accessory. Samples were ground with a mortar and pestle before analysis. 

#### 2.4.3. High-Performance Liquid Chromatography (HPLC)

The HPLC analysis of PET monomers and oligomers was adapted from Edwards et al., 2022 and Furukawa et al., 2018 [18,28]. The analysis of glycolysis products was conducted using an Agilent Technologies 1100 series HPLC equipped with a ZORBAX Eclipse Plus C18 (Rapid Resolution, 4.6 × 100 mm 3.5 Micron) column (Agilent Technologies, Santa Clara, CA, USA). The mobile phase was 70% MilliQ water, 20% acetonitrile, and 10% formic acid at a flow rate of 1.0 mL min^−1^, with a column temperature of 40 °C. 

HPLC samples were obtained by diluting a set amount of each glycolysis product in dimethyl sulfoxide (DMSO) to produce a 5 mM concentration (0.254 g of product diluted in 2 mL DMSO) and filtered through a 0.22 μm PES syringe filter. A TPA internal standard was added to each sample to produce a 5 mM concentration of TPA. Internal response control samples were created using a known amount of TPA and BHET (2 mM BHET, 5 mM TPA in 1 mL DMSO), and the internal response factor was calculated using Equation (3):

(3) $$Internal\;Response\;Factor\;\left(IRF\right)=\frac{\left[S_{IS}\right]\times \left[C_{BHET}\right]}{[S_{BHET}]\times \left[C_{IS}\right]}$$

where *S_IS_*, *S_BHET_*, *C_IS_*, and *C_BHET_* refer to the signal area of the internal standard, the signal area of *BHET*, the concentration of the internal standard (mM), and the concentration of *BHET* (mM), respectively. The concentration of *BHET* was then calculated using Equation (4):

(4) $$BHET\;concentration\;\left(\mathrm{mM}\right)=\frac{\left[C_{IS}]\times [S_{BHET}]\times [IRF\right]}{\left[S_{IS}\right]}$$

where *IRF* is the internal response factor calculated in Equation (3). Experimentally, *BHET* was observed at 2.2 min.

#### 2.4.4. Powder X-ray Diffraction (PXRD)

Powder X-ray diffraction was used to determine the composition of the glycolysis products and catalyst. PXRD spectra were obtained with a Scintag XDS-2000 powder X-ray diffractometer using Cu Kα irradiation (λ = 0.154 nm). Samples were ground with a mortar and pestle before analysis. Data analysis was performed on the Match! software version 14.1.25024 and all reference patterns were obtained from the Crystallography Open Database [29,30,31,32,33,34,35].

#### 2.4.5. Single-Crystal X-ray Diffraction (SCXRD)

Single-crystal X-ray diffraction was used to determine the crystal structure of the crystallized glycolysis product. Data were collected using a Rigaku XtaLAB mini II benchtop diffractometer, and visualization was performed in Mercury [36].

#### 2.4.6. Inductively Coupled Plasma Spectrometry (ICP)

Approximately 200 mg of OPA was weighed into a 30 mL Savillex Teflon beaker. Repeated additions of concentrated nitric acid (2 mL) and hydrogen peroxide (0.5 mL) were applied to the sample and allowed to react uncapped. Once the reaction slowed, the beaker was capped and heated on a hotplate at 120 °C. The sample was checked periodically, and additional aliquots were added until complete digestion was achieved. Mixed element standards were prepared from single element standards purchased from Inorganic Ventures. Calibration standards were created from the mixed element standard by serial dilution.

Lower-concentration analytes (Ag, Cr, Mo, Ni, Sn and Ti) were analyzed on a Thermo Scientific (Waltham, MA, USA) i-CAP RQ ICP-MS operated in both standard (Ag, Mo, Ni, Sn, and Ti) and KED (Cr) modes. Higher-concentration analytes (Al, Ca, Cu, Fe, K, Mn, Na, and Zn) were analyzed on a Spectro Arcos II ICP-OES operated in side-on configuration. Both ICP-MS and ICP-OES samples were analyzed at a 10-fold dilution of the digested sample.

### 2.5. Microbial BHET Degradation

BHET biodegradation was conducted using a consortium of 5 bacterial strains first reported by León-Zayas et al., 2019 [37]. Bacterial cultures of the full consortium consisted of 0.2% (*w*/*v*) BHET or glycolysis product (0.06 g of BHET or glycolysis product in 30 mL liquid carbon-free basal medium (LCFBM), for a 7.86 mM concentration) and 0.05% (*w*/*v*) yeast extract in 250 mL bacterial culture flasks with rubber-fitted lids. The 2-week and 10-week experimental cultures were inoculated with 1% (*v*/*v*) of the normalized OD_600_ consortium initially, and then also at each 2-week time point for the 10-week culture. The consortium contained equal quantities of each of the five bacterial species. Experimental samples that were inoculated with the consortium were created in triplicate, along with triplicate negative controls without any bacteria. Cultures were incubated at 30 °C, and culture media were tested every 2 weeks using HPLC quantitative analysis. 

### 2.6. HPLC Analysis of Microbial BHET Degradation

BHET degradation was quantified within each culture, with sample preparation and HPLC data analysis optimized. Culture media and DMSO were mixed in a 1:4 ratio (200 μL to 800 μL, for a 1 mL sample) and filtered through a 0.22 μm PES syringe filter to produce each HPLC sample. A 5 mM TPA internal standard was added to each sample for quantitative analysis, and internal response control factors were used to determine the BHET concentration in each microbial culture. Samples were analyzed using the same method and instrument as mentioned above. Experimentally, BHET, MHET, and TPA were observed around 2.2, 1.8, and 1.4 min, respectively.

### 2.7. Degradation Rate Calculation

The biodegradation rate was calculated by comparing the BHET concentrations of the uninoculated negative controls to the inoculated experimental samples. Since cultures were grown in liquid media, some BHET hydrolysis also naturally occurred, degradation that was independent of the bacteria. The biodegradation rate beyond hydrolysis (*B*_1_) tested the increased efficiency of the consortium through the BHET concentration (*C*_i_, i = week X inoculated sample, week X uninoculated sample, or week 0 uninoculated sample BHET concentration, where X is some number of weeks greater than 0) and was calculated using Equation (5):

(5) $$B_{1}\left(\%\right)=(1-\frac{C_{Week\;X\;inoculated\;sample}}{C_{Week\;X\;unioculated\;sample}})\times 100$$

Similarly, the biodegradation rate (*B*_2_), including biodegradation and hydrolysis, was calculated using Equation (6):

(6) $$B_{2}\left(\%\right)=(1-\frac{C_{Week\;X\;inoculated\;sample}}{C_{Week\;0\;unioculated\;sample}})\times 100$$

The total PET degradation rate of the two-step process, including the glycolysis yield (*G*_i_, where i = crystalline or powder solid product yield) and consortium biodegradation rate (*B*_i_, where i = crystalline or powder solid biodegradation rate), was calculated using Equation (7):

(7) $$Total\;PET\;Degradation\;Rate\;\left(\%\right)=\left(G_{Crystalline}\times B_{Crystalline}\right)+\left(G_{Powder\;Solid}\times B_{Powder\;Solid}\right)$$

## 3. Results and Discussion

### 3.1. PET Glycolysis

#### 3.1.1. OPA Characterization

OPA was produced as described in the Materials and Methods. The metal-ion and organic composition of the OPA product was characterized by ICP, FTIR-ATR, and PXRD, respectively, to determine the catalytic mechanism of PET glycolysis to form BHET. 

ICP analysis confirmed the elemental composition of the OPA catalyst, providing the following results: K (117 ± 1.1 mg/g), Ca (86.5 ± 0.71 mg/g), Cu (1.24 ± 0.02 mg/g), Na (410 ± 3.7 µg/g), Zn (280 ± 4.3 µg/g), Fe (160 ± 7.5 µg/g), Mn (70.8 ± 3.72 µg/g), Al (54.7 ± 3.65 µg/g), Ti (22.6 ± 0.21 µg/g), Ni (4.24 ± 0.06 µg/g), Sn (3.71 ± 0.02 µg/g), Mo (1.29 ± 0.01 µg/g), Cr (0.677 ± 0.019 µg/g), and Ag (0.510 ± 0.003 µg/g). These results align with the analytical observations made about the OPA composition in Changmai et al., 2019 and Lalhmangaihzuala et al., 2021 [12,38].

**Figure 1:**
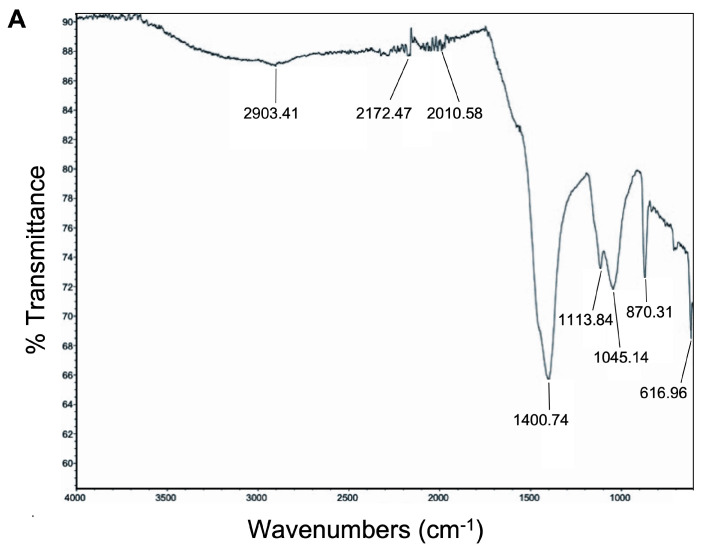

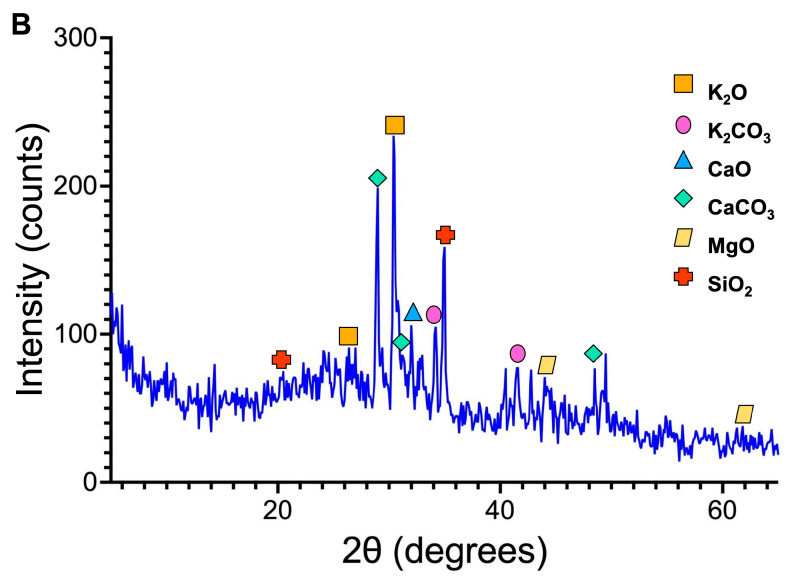
(**A**) FT-IR full spectrum (4000 to 600 cm^−1^) of OPA catalyst. The spectrum was taken with a solid sample using a diamond ATR accessory. (**B**) Powder X-ray diffraction pattern of OPA catalyst.

The FT-IR spectra revealed the presence of carbonate ions, as the absorption peaks at 1400, 1045, and 870 cm^−1^ were assigned to the C-O stretching and bending frequency (Figure 1A [38,39]. The absorbance peak at 616 cm^−1^ corresponded to the Ca-O stretching frequency. XRD analysis was performed to explore the crystalline components of the catalyst (Figure 1B). Peaks for K_2_O and K_2_CO_3_ were observed at 2θ = 24.15, 41.50, 34.14, 30.45° (COD ID no. 9001563 and 9010971). Peaks for CaO and CaCO_3_ were observed at 2θ = 32.00, 29.40, 31.25, 48.55° (COD ID no. 1011095 and 1010962). Peaks for MgO and SiO_2_ were observed at 2θ = 42.80, 62.23, 21.55, 35.64° (COD ID no. 1011116 and 1010954). 

**Figure 2:**
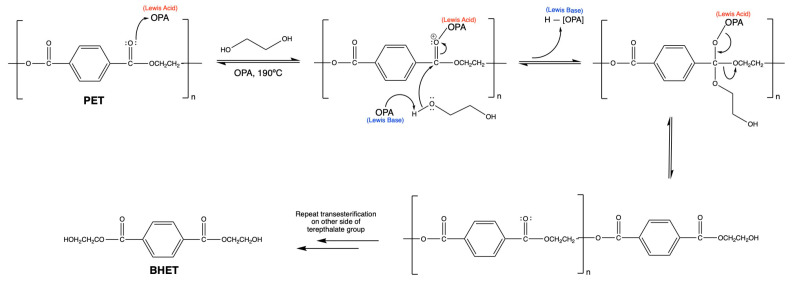
**Proposed arrow pushing mechanism for OPA-catalyzed glycolysis of PET.** Because the OPA catalyst contains both positively and negatively charged ions (through the high concentrations of metal oxides and carbonates), it can act as a Lewis acid and a Lewis base, which is indicated according to the formal charge of the catalyst.

Based on the characterization results and previous studies, it is predicted that OPA can catalyze PET depolymerization through its high concentration of metal oxides and carbonates, which can act as both Lewis acids and bases [7,12,38]. The proposed mechanism is shown in Figure 2. In the glycolysis of PET, OPA first acts as a Lewis acid catalyst using the cationic metal centers of the metal oxides and carbonates. This occurs by bonding with the carbonyl oxygen atom to remove the electron density and to activate the carbonyl for a nucleophilic attack. Using the anionic oxygen or carbonate centers in the metal oxides and carbonates, OPA then acts as a Lewis base to deprotonate ethylene glycol and increase the molecule’s nucleophilicity. The carbonyl carbon undergoes a nucleophilic attack by one of the oxygen atoms on ethylene glycol, and a tetrahedral intermediate is formed. Electrons from the former carbonyl oxygen regenerate the carbonyl, and this results in transesterification as the original ester bond is cleaved, which breaks the polymer chain backbone. Once transesterification occurs on both ester groups of the terephthalate group, BHET is produced, with ethylene glycol being reformed as a byproduct (Figure 2). 

#### 3.1.2. Yield and Characterization of the Glycolysis Products

PET depolymerization was performed via refluxing post-consumer PET flakes, with the OPA catalyst and ethylene glycol, and then isolating the product through crystallization. BHET was synthesized in a 90.2% yield, and 100% PET conversion was achieved. Products were characterized through NMR (1D ^1^H and ^13^C), FTIR-ATR, HPLC, SCXRD, and PXRD. 

Due to the nature of the two-step degradation process, any byproducts of the glycolysis reaction were collected to explore the biocompatibility with the bacterial consortium. Every glycolysis reaction that was run, regardless of the crystallization and workup method used, yielded two products: transparent, orange, needle-like crystals and a pale brown powder-like solid, the latter of which was separated during the reaction workup before crystallization occurred. The workup and crystallization methods used after the reaction had a substantial effect on the amount of each type of product produced. Since the crystalline product was more consistent with recrystallized BHET compared to the powder-like solid, the workup procedure was varied to find the optimal conditions to produce a large amount of the crystalline solid. As seen in Table 1, the agitation method produced a significantly larger amount of crystalline product, 78.4%, and maximized the total yields of both products. 

**Figure 3:**
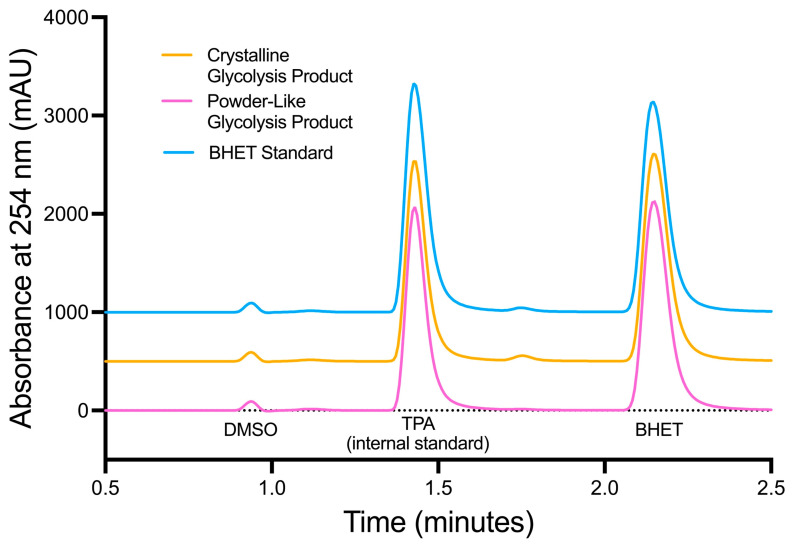
**HPLC chromatograms of glycolysis products and BHET standard.** A 5 mM TPA internal standard was added to each sample, observed at 1.4 min, and BHET was observed at 2.2 min. The dotted line indicates zero absorbance.

**Figure 4:**
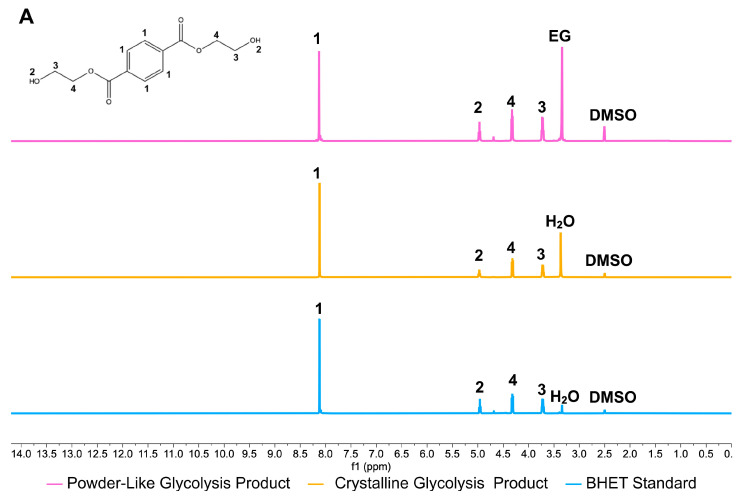

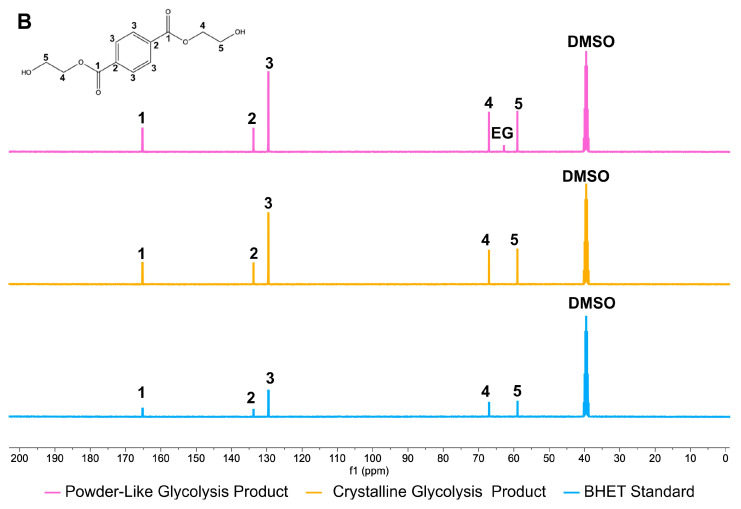
**NMR overlaid spectra for PET glycolysis products.** (**A**) ^1^H NMR full spectrum from 0 to 14 ppm of powder-like glycolysis product (top), crystalline glycolysis product (middle), and BHET standard (bottom). (**B**) ^13^C NMR full spectrum from 0 to 200 ppm of powder-like glycolysis product (top), crystalline glycolysis product (middle), and BHET standard (bottom). All spectra are referenced to DMSO-d6.

When tested through HPLC, both the crystalline and powder glycolysis products were observed to have a large peak at 2.2 min, which was consistent with the BHET standard retention time (Figure 3). Initially, it was thought that the powder-like solid was primarily composed of BHET oligomers. However, HPLC analysis showed no peaks after 2.2 min, at which oligomers would elute. When tested through NMR, all signals that were present in the BHET standard were displayed in the spectra for both glycolysis products (Figure 4). A water trace impurity in the ^1^H NMR spectra of the crystalline product and an ethylene glycol trace impurity in the ^13^C NMR spectra of the powder-like product were observed. Through this analysis, the powder-like solid product was likely unable to crystallize, in part due to this trace amount of ethylene glycol left in the solid. Full NMR spectra of the glycolysis products can be seen in Figures S1, S2, S4 and S5. FTIR-ATR spectroscopy of both products was consistent with the BHET standard, as all expected absorption peaks from the BHET standard were present in the spectra of both products (Figures S3 and S6). 

The PET glycolysis reaction procedure outlined here uses relatively mild reaction conditions and reagents compared to many studies in the literature [6]. While it is becoming more common to run glycolysis reactions under atmospheric pressure, the use of biomass-derived heterogeneous catalysts, such as OPA, is not standard practice, as the emergence of these types of catalysts is relatively recent and they have not been studied extensively [6,40]. The use of the OPA catalyst was chosen for this work because it was readily available as bio-waste and likely to be biocompatible with the consortium. In turn, the glycolysis products were not expected to be pure but still biocompatible, so further recrystallization and purification of the products was not necessary. The reaction procedure allows for a modified one-pot biochemical degradation process, as outlined in Kim et al., where, instead of the entire glycolysis slurry being used for biodegradation, solid products are separated during workup for bacterial degradation [22]. This was primarily done to quantify BHET bacterial degradation, as BHET byproduct accumulation after biodegradation would not be observed since the consortium would theoretically completely degrade all byproducts [18].

### 3.2. Biodegradation of Synthesized BHET

Glycolysis product biodegradation was performed to determine the biocompatibility of the synthesized product and the efficiency of degradation by a five-species bacterial consortium. While the crystalline glycolysis product was confirmed to be BHET (Figure 3), it was unknown whether the powder-like glycolysis product would undergo the same degradation mechanism due to the presence of ethylene glycol. The bacterial consortium could utilize ethylene glycol as a carbon source, noted in Roberts et al., 2020, so it was predicted that the powder-like solid product also would be biocompatible [17]. 

Because the bacterial cultures were grown in liquid media, primarily composed of water, BHET hydrolysis could occur to degrade the compound without inoculation. To explore the increased efficiency of biodegradation compared to hydrolysis alone, the degradation results of inoculated cultures were compared to those of uninoculated negative controls at each time point. In addition, the total degradation of BHET, including both biodegradation and hydrolysis, was measured by comparing the inoculated cultures at each time point to the uninoculated negative controls from time point zero. The latter approach accounted for any initial hydrolysis that occurred upon the addition of BHET to the culture media but not hydrolysis over time, which was reflected in the total degradation percentage.

**Figure 5:**
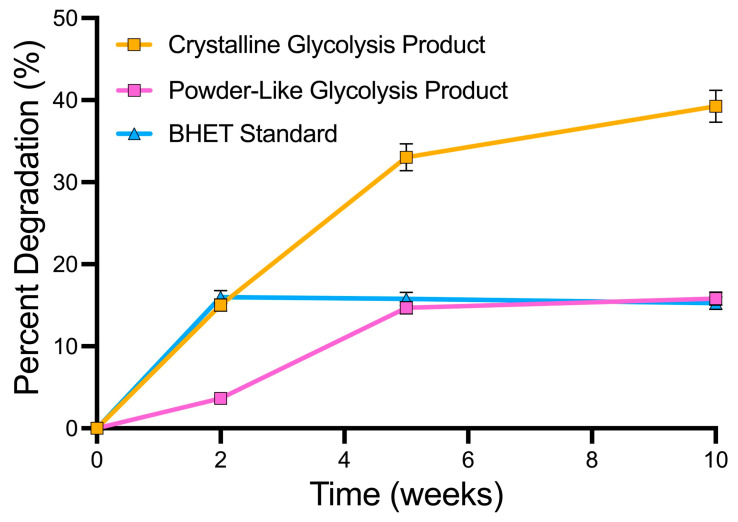
**Consortium biodegradation of BHET and glycolysis products over time.** The full consortium was grown on 0.2% (*w*/*v*) BHET, crystalline glycolysis product, or powder-like glycolysis product as sole carbon source in LCFBM. Cultures were incubated statically at 30 °C. Degradation was measured every 2 to 3 weeks through HPLC, and concentration differences were measured using an internal standard and uninoculated negative controls. All experiments were performed in triplicate. Error bars indicate standard error.

**Figure 6:**
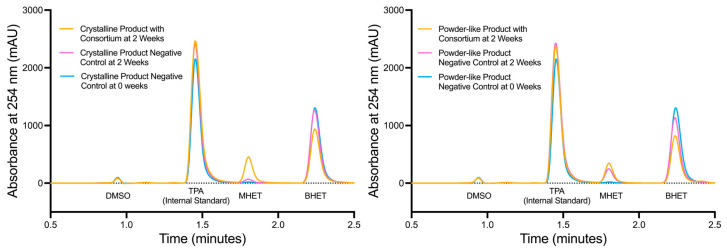
**HPLC chromatogram overlay of BHET degradation.** HPLC overlaid chromatograms of crystalline (**left**) and powder-like (**right**) glycolysis product degradation. A 5 mM TPA internal standard was added to each sample, observed at 1.4 min, and BHET was observed at 2.2 min. BHET signal area was compared to the internal standard signal area as part of the calculation for BHET concentration, so BHET peak height and area should be analyzed comparatively to TPA peak height and area. Samples were diluted in a 1:4 ratio of culture media to DMSO. The dotted line indicates zero absorbance.

As seen in Figure 5, there was significant degradation of the glycolysis products when comparing the inoculated and uninoculated cultures, indicating that the consortium can degrade OPA-catalyzed glycolysis products to a greater extent than BHET hydrolysis alone. When the glycolysis products were used as the sole carbon source without any supplemental nutrition, the 10-week degradation rate was 39.24% for the crystalline glycolysis product, 15.82% for the powder-like glycolysis product, and 15.26% for the BHET standard (Figure 5). Another degradation assay using yeast extract-supplemented LCFBM was performed to determine if the consortium degradation rate increased with additional nutrition (Figure 6). Here, we found that the 2-week degradation rate was 19.81% for the crystalline glycolysis product, 12.29% for the powder-like glycolysis product, and 8.69% for the BHET standard (Table 2). When including hydrolysis into the degradation rate, the 2-week degradation rate for all three compounds using yeast-extract-supplemented media increased to over 60% (Table 2). 

### 3.3. Effect of BHET Solid Structure on Biodegradation

Because of the varied biodegradation rates observed between the powder-like glycolysis product, the crystalline glycolysis product, and the BHET standard, it can be said that biodegradation is affected by the type of BHET polymorph or solvate used as a carbon source. Depending on the workup and crystallization method used after the reaction, different BHET polymorphs and solvates can form. Through the characterization of these products using NMR, SCXRD, and PXRD, the rate of the biodegradation mechanisms can be further understood. 

The crystalline glycolysis product was confirmed to be BHET with a small water trace impurity through NMR, IR, and HPLC analysis. When tested through SCXRD, it was observed that the crystal was a known BHET polymorph, first cited in Scé et al., 2019 (Figure S7) [41]. The BHET polymorph has a space group of P-1, indicating no symmetry within the unit cell, and its crystallographic unit cell contains 11 molecules. The crystalline structure is primarily held together by intermolecular hydrogen bonds from the ester and alcohol heteroatoms and offset face-to-face pi stacking between the arenes within the terephthalate group. 

**Figure 7:**
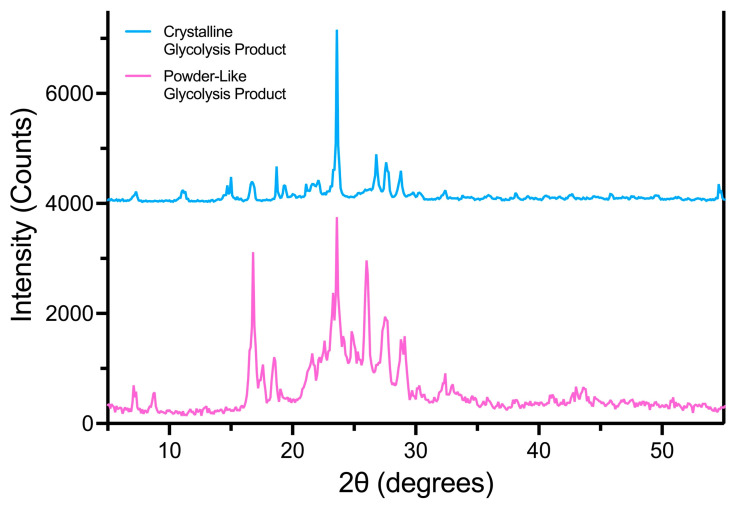
Powder X-ray diffraction pattern overlay of crystalline and powder-like glycolysis products.

Both glycolysis products and the BHET standard were analyzed through PXRD to explore the crystalline components that composed the products. The powder-like solid glycolysis product was characterized as a BHET EG solvate, due to trace ethylene glycol found during ^13^C NMR analysis (Figure S5). PXRD analysis of the powder-like solid product showed the presence of crystalline metal components such as CaCO_3_, MgO, and CaO, likely contributing to the product being unable to crystallize during the reaction workup (Figure 7). Nevertheless, the HPLC, NMR, and IR analyses of the crystalline and powder-like BHET indicated the chemical similarities between the two products (Figure 6 and Figures S1–S6). 

The microbial degradation of solids containing metal components, such as metal oxides and carbonates in the case of the powder-like glycolysis product, has been studied extensively, to better understand the effects of the metal components in the soil matrix on bacteria [42]. There is evidence that magnesium and calcium ions increase the activity of lignocellulolytic enzymes, which can degrade lignin and have very similar degradation mechanisms to PET-degrading enzymes [43]. It can be hypothesized that the PET-degrading microbial consortium used in this experiment could also have increased degradation activity due to the addition of metal oxides. Nevertheless, more research is needed to elucidate the influence of metal-containing components in solids on the enzymatic biodegradation rates of plastic-degrading bacteria.

### 3.4. Efficacy of the Two-Step Degradation Process

The consortium degraded BHET and, more notably, was able to degrade the glycolysis products without further purification after the OPA-catalyzed reaction. The biodegradable glycolysis products were produced in a 90.21% yield, so the combined downstream biodegradation of these products has large implications for sustainable plastic recycling. If considering both steps, and not including additional degradation due to hydrolysis, the two-step process can degrade post-consumer PET by 32.63% over 10 weeks (if using the glycolysis products as the sole carbon source during biodegradation) and 16.98% in 2 weeks (if using yeast-supplemented LCFBM). When including the hydrolysis of PET monomers during biodegradation, the two-step process can degrade post-consumer PET by 56.50% in 2 weeks. A comparison of these numbers can be seen in Table 3. 

It should also be noted that most microbial degradation studies use low-crystallinity PET film, which ranges from 2% to 5% crystalline, rather than post-consumer PET, which is approximately 20% crystalline [21,26,44,45,46]. The consortium degradation, and most enzymatic degradation, works best with less crystalline polymers due to the increased accessibility of bonds and weakened bond strength, so the two-step degradation rate reported here is a noteworthy result [47,48,49,50]. Our results are comparable to those of Ricarte et al., 2021, who also used a two-step process to depolymerize highly crystalline PET from plastic waste [25]. With urea/Zn(OAc)_2_·2H_2_O as the catalyst for glycolysis, PET was converted to BHET at 100% efficiency, and then the CALB enzyme was used to convert BHET to TPA. The overall yield of the two-step process was 57%, similar to our results using whole bacteria (Table 3).

## 4. Conclusions

The two-step process of PET depolymerization followed by whole bacterial consortium biodegradation is an efficient method for the complete degradation of post-consumer, highly crystalline PET plastic. The chemical glycolysis of PET with an OPA catalyst and ethylene glycol as a solvent produced biocompatible BHET-containing products in a 90.21% yield and was able to fully depolymerize PET. The glycolysis products were subsequently degraded by 62.63% in 2 weeks through bacterial consortium biodegradation. The combined two-step PET degradation rate was 56.50% in 2 weeks. Thus, chemical depolymerization coupled with bacterial degradation is an effective and sustainable way to combat PET pollution and mitigate the effects of environmental fallout without using costly or harmful reagents or procedures. 

## Figures and Tables

**Table 1:** Influence of workup and crystallization methods used after glycolysis reaction on product yield.

Workup Method Used	Yield of Crystalline Product (%) ^1^	Yield of Crystalline and Water-Insoluble Product (%) ^1^
Concentration ^2^	16.8	86.8
Agitation ^3^	78.4	90.2
Concentration and Agitation ^4^	26.6	85.3

^1^ Yield was calculated using Equation (2). ^2^ Reaction mixture was quickly vacuum-filtered with 60 °C H_2_O after reaction and filtered again to separate water-insoluble product after cooling to room temperature. The mixture was then concentrated to half of its original volume and refrigerated overnight at 4 °C for crystallization. ^3^ Reaction mixture was quickly vacuum-filtered with 60 °C H_2_O after reaction, stirred vigorously for 45 min, filtered again to separate water-insoluble product, and cooled overnight for crystallization. ^4^ Reaction mixture was quickly vacuum-filtered with 60 °C H_2_O after reaction, stirred vigorously for 45 min, and filtered again to separate water-insoluble product. The mixture was then concentrated to half of its original volume and refrigerated overnight at 4 °C for crystallization.

**Table 2:** BHET biodegradation rate of glycolysis products and BHET standard with and without hydrolysis included.

Carbon Source	2-Week Yeast-Extract-Supplemented Degradation Rate Including Hydrolysis (%) ^1^	2-Week Yeast-Supplemented Degradation Rate without Hydrolysis (%) ^2^
Crystalline glycolysis product	62.80	19.81
Powder-like glycolysis product	61.55	12.29
BHET standard	61.32	8.69

^1^ Biodegradation rates were calculated using Equation (5). ^2^ Biodegradation rates were calculated using Equation (6).

**Table 3:** Comparison of degradation assay conditions’ impact on biodegradation and two-step degradation rates.

Degradation Assay	Biodegradation Rate of Glycolysis Products (%) ^1^	Two-Step Degradation Rate (%) ^1^
10-week degradation assay ^2^	35.30	32.63
2-week degradation assay not including hydrolysis	18.82	16.98
2-week degradation assay including hydrolysis ^3^	62.63	56.50

^1^ The average biodegradation rate of glycolysis products was calculated by averaging the degradation rates of the two products with respect to the ratio of products yielded during the glycolysis reaction. The two-step degradation rate was calculated through Equation (7). ^2^ 10-week degradation assay was conducted with the glycolysis products as the sole carbon source, whereas the 2-week degradation assay introduced yeast supplementation to lessen the stress of a non-preferential carbon source. The 10-week degradation assay includes hydrolysis in biodegradation rates. ^3^ The 2-week degradation assay rates (listed in rows 2 and 3) are calculated from the same data, where the entry in row 2 controls for hydrolysis in biodegradation calculations, whereas the entry in row three does not (and thus includes hydrolysis rates within biodegradation data).

## Data Availability

The data are contained within the article.

## Supplementary Materials

The following supporting information can be downloaded at: https://www.mdpi.com/article/10.3390/bioengineering10111253/s1, Figure S1: ^1^H NMR Spectrum of Crystalline Glycolysis Product, Figure S2: ^13^C NMR Spectrum of Crystalline Glycolysis Product, Figure S3: FTIR Spectrum of Crystalline Glycolysis Product, Figure S4: ^1^H NMR Spectrum of Powder-Like Glycolysis Product, Figure S5: ^13^C NMR Spectrum of Powder-Like Glycolysis Product, Figure S6: FTIR Spectrum of Powder-Like Glycolysis Product, Figure S7: SCXRD of Crystalline Glycolysis Product.
