# Supplementary material for: Two-Step Chemo-Microbial Degradation of Post-Consumer Polyethylene Terephthalate (PET) Plastic Enabled by a Biomass-Waste Catalyst
Source: Bioengineering (Basel). 2023 Oct 26;10(11):1253. doi: 10.3390/bioengineering10111253 (PMC10669257; doi:10.3390/bioengineering10111253)
Supplement: Supplementary file 1 [file bioengineering-10-01253-s001.zip › bioengineering-2610116-supplementary.pdf]

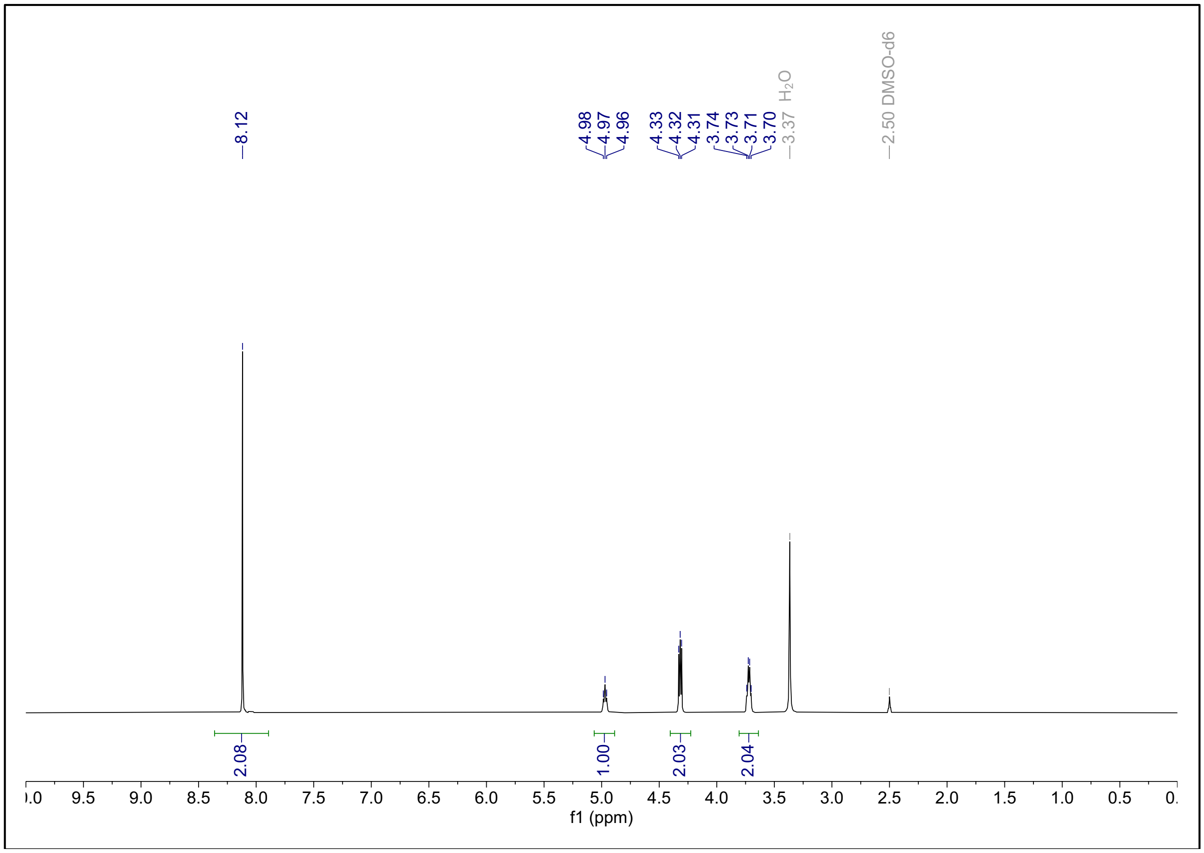

Figure S1. ^{1}H NMR full spectrum from 10 to 0ppm of crystalline glycolysis product. The spectrum is referenced to DMSO-*d6*. A water impurity can be seen at 3.4 ppm.

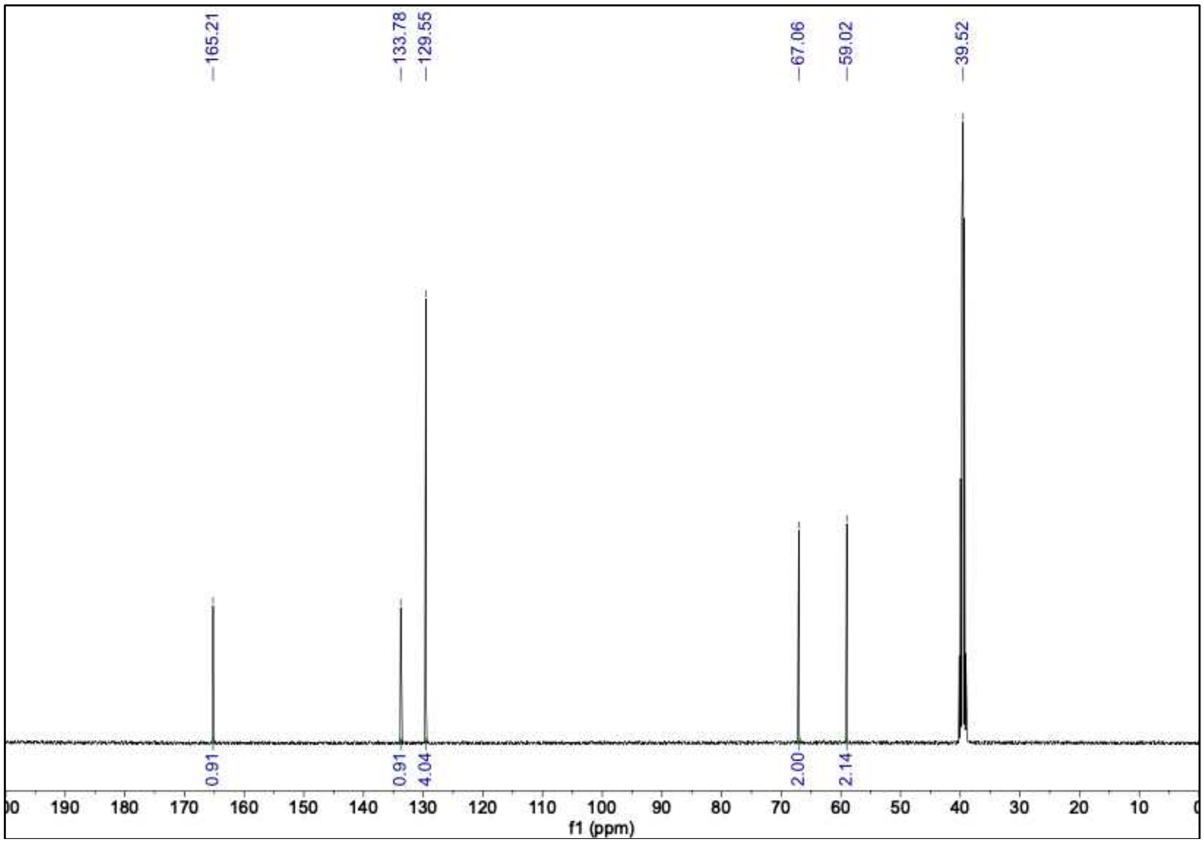

Figure S2. ^{13}C NMR full spectrum from 200 to 0 ppm of crystalline glycolysis product. The spectrum is referenced to DMSO-*d6*.

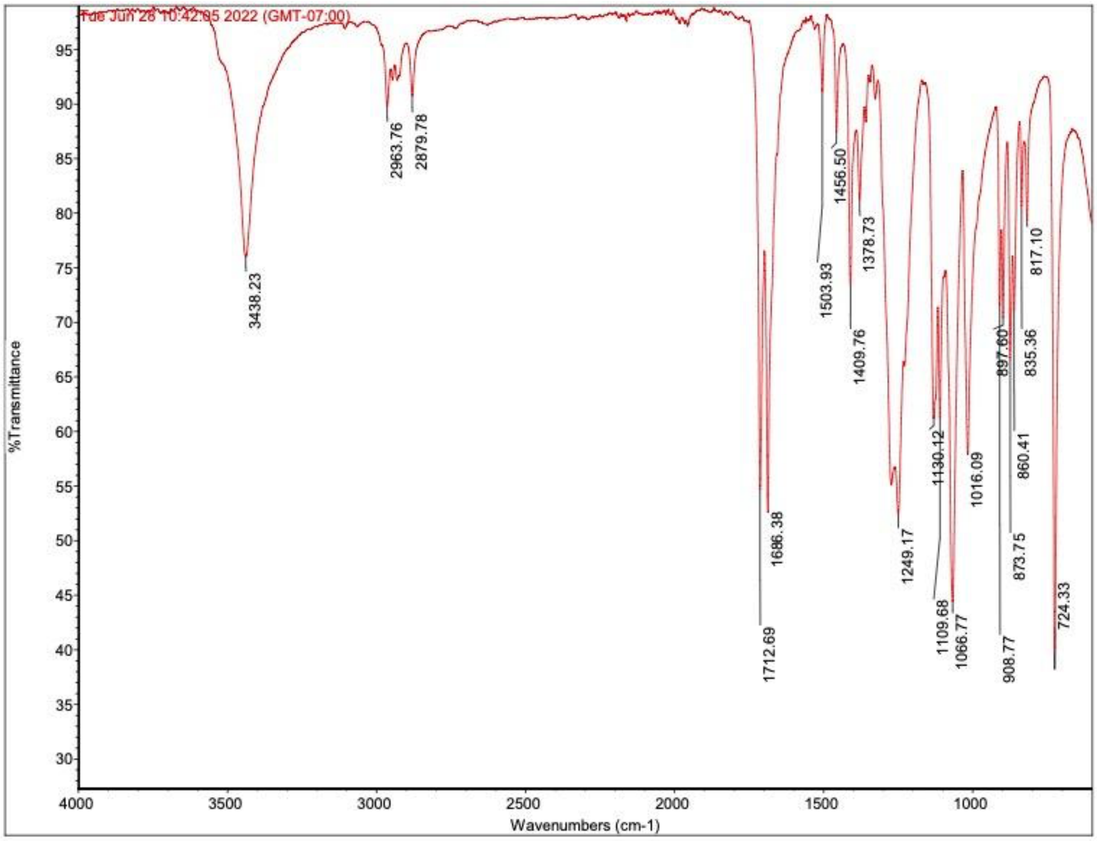

Figure S3. IR full-spectrum (4000 to 600 cm^{-1}) of crystalline glycolysis product. The spectrum was taken with a solid sample using a diamond ATR accessory.

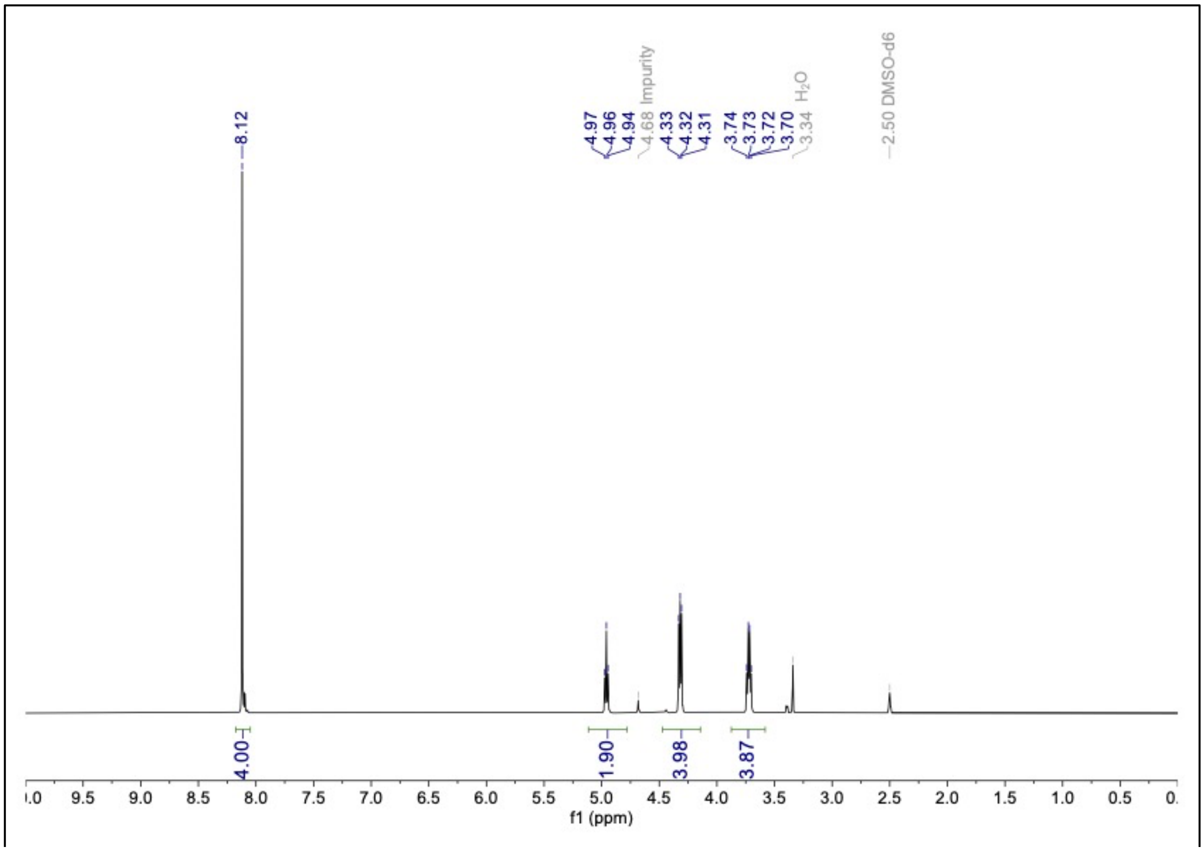

Figure S4. ^{1}H NMR full spectrum from 10 to 0 ppm of powder-like glycolysis product. The spectrum is referenced to DMSO-*d6*. An ethylene glycol impurity can be seen at 3.4 ppm and an unknown impurity can be seen at 4.7 ppm.

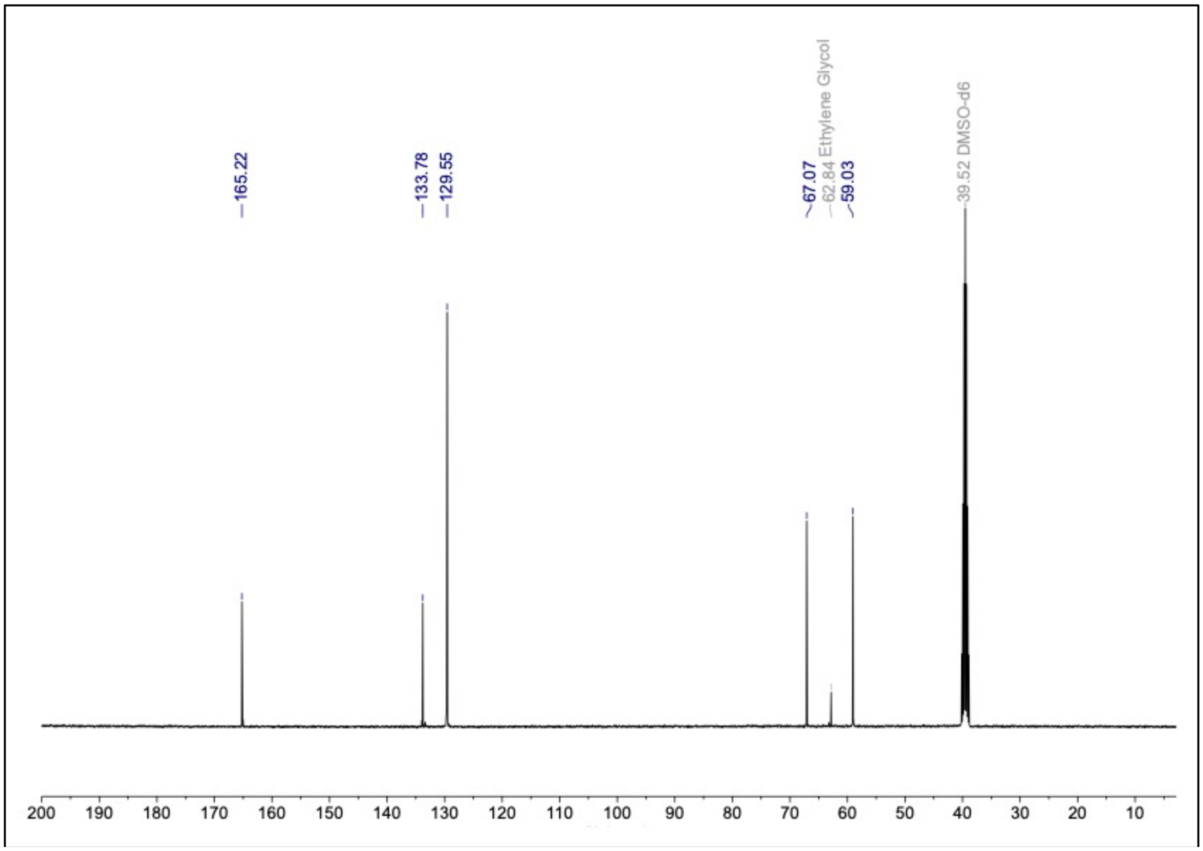

Figure S5. ^{13}C NMR full spectrum from 200 to 0 ppm of powder-like glycolysis product. The spectrum is referenced to DMSO-*d6*. An ethylene glycol impurity can be seen at 62.8 ppm.

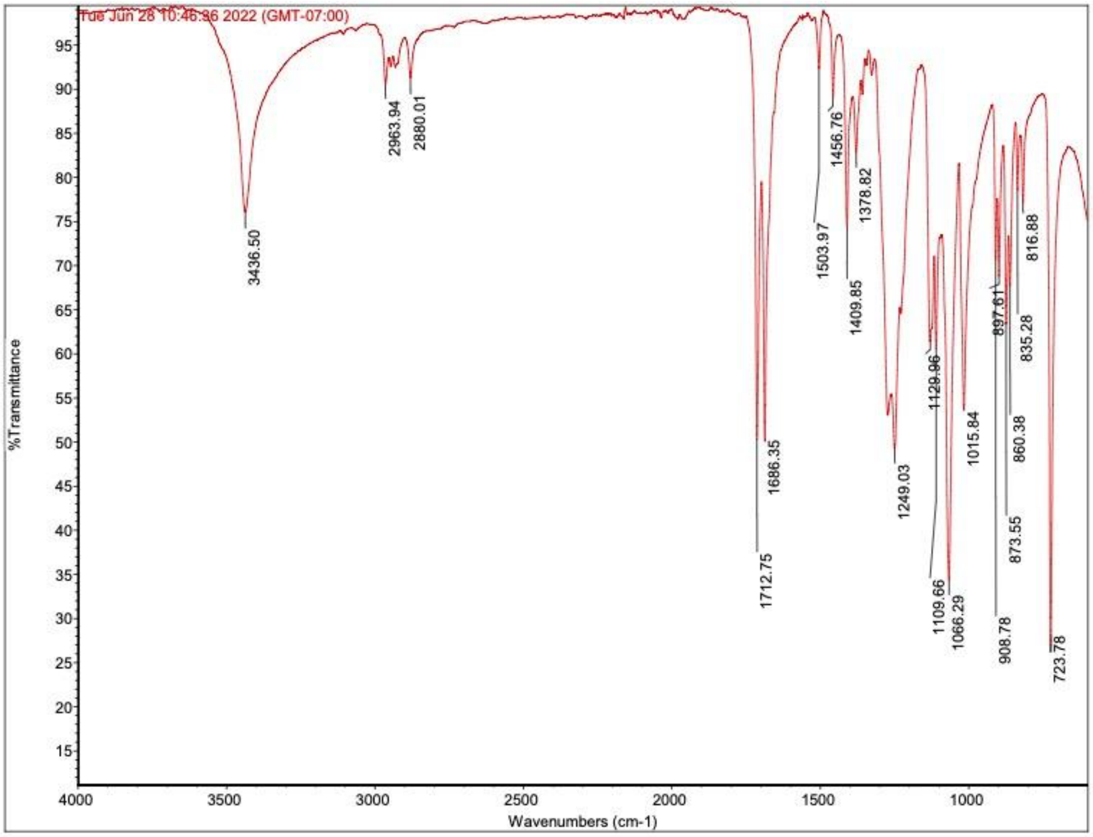

Figure S6. IR full-spectrum (4000 to 600 cm^{-1}) of powder-like glycolysis product. The spectrum was taken with a solid sample using a diamond ATR accessory.

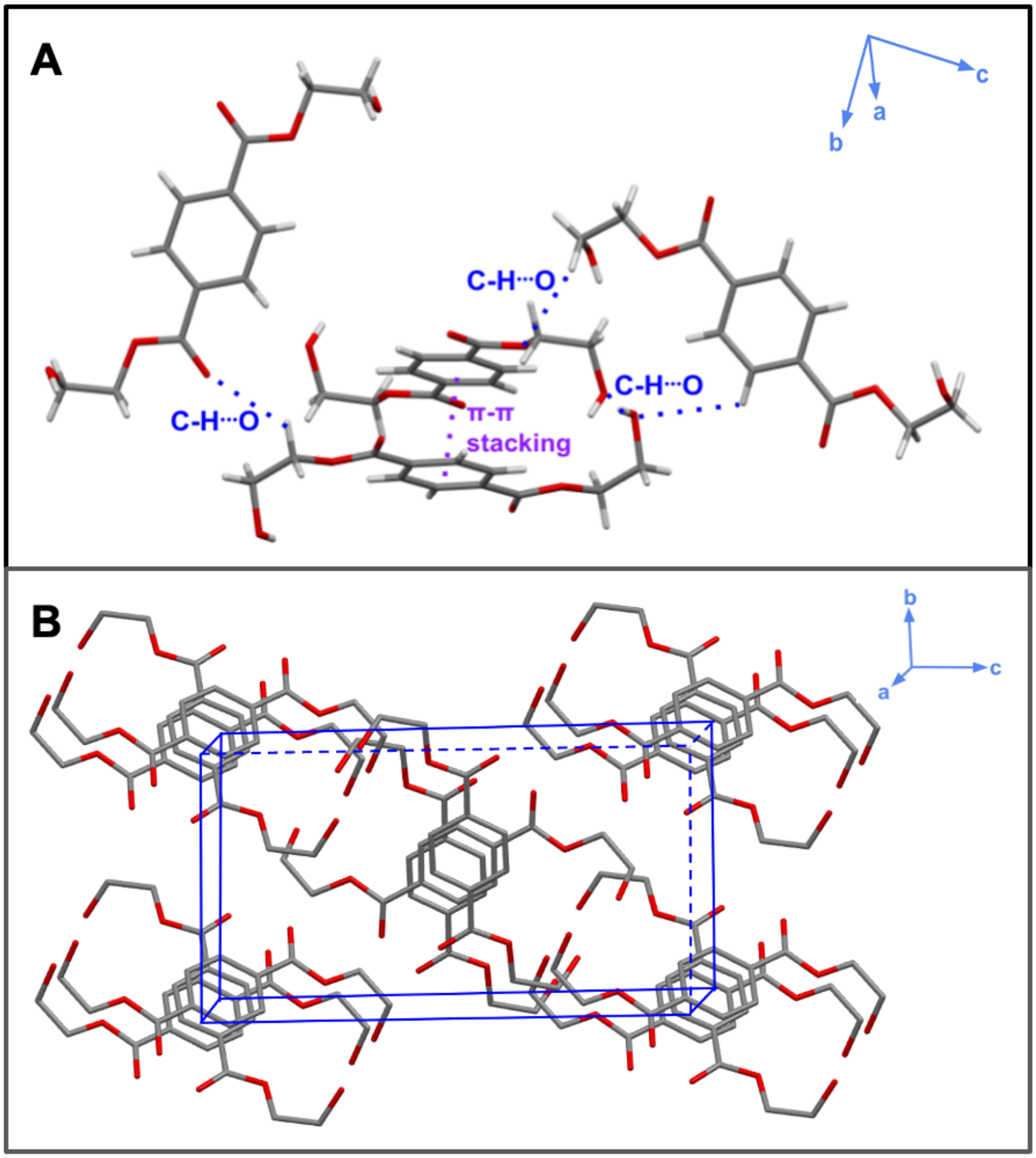

Figure S7. SCXRD of crystalline BHET product. **(A)** Representative short contact interactions (minimum = sum of vdW radii minus 5, maximum = sum of vdW radii plus 0.15) between molecules 0.5*0.5*0.5 packing structure. Interactions include C-H· · ·O (in blue) and π· · ·π (in purple) **(B)** 1*1*1 packing structure with unit cell borders in blue. Hydrogen atoms are omitted for clarity.
